# Perception, Action, and Roelofs Effect: A Mere Illusion of Dissociation

**DOI:** 10.1371/journal.pbio.0020364

**Published:** 2004-10-26

**Authors:** Paul Dassonville, Jagdeep Kaur Bala

**Affiliations:** **1**Department of Psychology and Institute of Neuroscience, University of OregonEugene, OregonUnited States of America

## Abstract

A prominent and influential hypothesis of vision suggests the existence of two separate visual systems within the brain, one creating our perception of the world and another guiding our actions within it. The induced Roelofs effect has been described as providing strong evidence for this perception/action dissociation: When a small visual target is surrounded by a large frame positioned so that the frame's center is offset from the observer's midline, the perceived location of the target is shifted in the direction opposite the frame's offset. In spite of this perceptual mislocalization, however, the observer can accurately guide movements to the target location. Thus, perception is prone to the illusion while actions seem immune. Here we demonstrate that the Roelofs illusion is caused by a frame-induced transient distortion of the observer's apparent midline. We further demonstrate that actions guided to targets within this same distorted egocentric reference frame are fully expected to be accurate, since the errors of target localization will exactly cancel the errors of motor guidance. These findings provide a mechanistic explanation for the various perceptual and motor effects of the induced Roelofs illusion without requiring the existence of separate neural systems for perception and action. Given this, the behavioral dissociation that accompanies the Roelofs effect cannot be considered evidence of a dissociation of perception and action. This indicates a general need to re-evaluate the broad class of evidence purported to support this hypothesized dissociation.

## Abstract 2

Paul Dassonville and Jagdeep Bala challenge a prominent hypothesis that proposes the existence of two separate visual systems within the brain, one creating perception and the other guiding action

## Introduction

Several anatomical, neurophysiological, clinical, and behavioral investigations of human subjects and nonhuman primates have provided evidence for two separate and dissociable cortical systems for visual processing. One of these systems—the ventral stream—resides in a swath of cortex that extends in an anteroventral direction from primary visual cortex to the temporal lobe. The second system resides in a dorsal stream that roughly extends from primary visual cortex to the parietal lobe. These ventral and dorsal systems were originally thought to be dedicated to the visual processing required to determine an object's identity and location, respectively (Ungerleider and Mishkin 1982). However, a more recent hypothesis suggests that both streams process information concerning object properties and locations, but that they do so for different purposes. In this revised model of the visual system (Milner and Goodale 1995), the ventral stream is presumably responsible for the formation of perceptual/cognitive representations of objects and events in the world, whereas the dorsal stream is responsible for guiding sensorimotor actions in response to those objects and events.

Much of the evidence for separate perception and action systems has come from behavioral studies of normal subjects. The general logic of these behavioral paradigms (e.g., Bridgeman et al. 1981, 1997; Aglioti et al. 1995; Daprati and Gentilucci 1997; Goodale and Murphy 1997; Haffenden and Goodale 1998) is as follows: An illusory stimulus is presented to the subject, who is required to report some characteristic of the stimulus (location, size, orientation, etc.) using perceptual (e.g., verbally compare the test object to some reference object) or sensorimotor means (e.g., make a movement to reach toward or grasp the test object). As a general finding, it seems that perceptual reports are more prone to illusions than are sensorimotor responses, suggesting that the systems are dissociable not only in terms of their cortical pathways, but also in terms of their processing capabilities.

**Figure 1:**
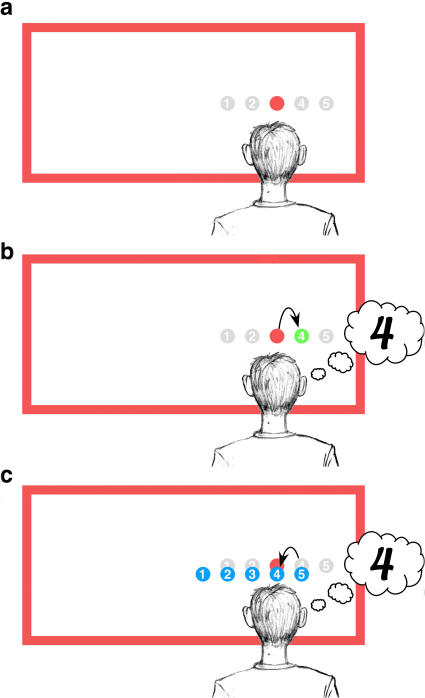
Schematic of the Induced Roelofs Effect (A) Example visual display (not drawn to scale) comprising a target (red circle) and a frame offset to the subject's left. Gray circles (unseen by subjects) represent the remembered positions of the items within the comparison array, centered on the subject's midline. (B) One possible mechanism for the inaccurate perceptual report of the target location, based on an illusory rightward shift of the perceived target location (green circle). (C) An alternative mechanism for the inaccurate perceptual report, based on a leftward shift of the memorized location of the comparison array (blue circles). Either mechanism (B or C) would result in the subject reporting the target to occupy the remembered location of item 4 in the comparison array.

As a specific example of this type of evidence, Bridgeman et al. (1997) tested the ability of subjects to indicate the location of a small visual target presented within an illuminated frame that was offset left or right from the subject's midline plane (Figure 1A). When asked to perceptually compare the location of the target with respect to an array of possible locations learned earlier, subjects reported the target to be in a location that was shifted in a direction opposite that of the frame—a perceptual phenomenon known as the induced Roelofs effect (Bridgeman et al. 1997; see also Roelofs 1935). In contrast, subjects could accurately guide the hand to the target's location, indicating that sensorimotor localization was immune to the illusion. These findings were cited as strong evidence for the existence of two distinct, differently abled visual systems for perception and action. However, Bridgeman et al. (1997) further demonstrated that not all actions were immune to the illusion; in particular, sensorimotor responses were prone to the induced Roelofs effect when subjects were required to point to the remembered location of the target after a delay of 4 s. As suggested by Bridgeman et al. (1997), this delayed sensorimotor effect could possibly be explained by a sensorimotor system that lacks a memory of its own and therefore must rely on the memory of the illusion-prone perceptual system to determine the goal of a movement directed toward a remembered target.

Although these findings are compatible with the hypothesis of two separate visual systems that are differentially affected by Roelofs illusion, it is important to consider the possibility of an alternative explanation. While it is true that both perceptual and sensorimotor measures within the task of Bridgeman et al. (1997) assayed the subjects' abilities to determine the location of the target, the two measures did so in markedly different ways. For the sensorimotor task, subjects could complete the task knowing only the location of the target within a body-centered or egocentric reference frame. In contrast, the perceptual task required the subjects to compare the location of the target relative to the locations of the items within the remembered comparison array. Given this, errors in the perceptual report could be due to either a misrepresentation of the target's position (Figure 1B) or a misrepresentation of the position of the remembered comparison array (Figure 1C). Whereas Bridgeman et al. (1997) concluded that the target is perceptually mislocalized in a direction opposite the frame offset, an inaccurate perceptual report could equally be due to a memory of the comparison array that is shifted in the same direction as the frame. The studies presented here were designed to test this alternative hypothesis for the mechanism underlying the induced Roelofs effect, and to critically examine the apparent dissociation of perception and action related to the illusion. We first replicate the findings of Bridgeman et al. (1997), using saccadic eye movements rather than a pointing task. We then test subjects' memory for the comparison array and show that it is biased by the offset frame in a way that can completely account for the perceptual illusion. A subsequent experiment demonstrates that this distortion of remembered visual space occurs when the brain, faced with an impoverished visual environment, incorrectly uses the location of the frame as a cue to establish an egocentric reference map whose origin (the apparent midline) is transiently biased toward the direction of the frame. Furthermore, movements guided within this same distorted reference map are shown to be accurate, given that the errors of target localization will be cancelled by subsequent errors of motor guidance. Thus, the perceptual and sensorimotor effects of the Roelofs illusion can be mechanistically explained without requiring the existence of separate neural processing streams for perception and action.

## Results/Discussion

### Perceptual and Sensorimotor Effects of the Illusion

**Figure 2:**
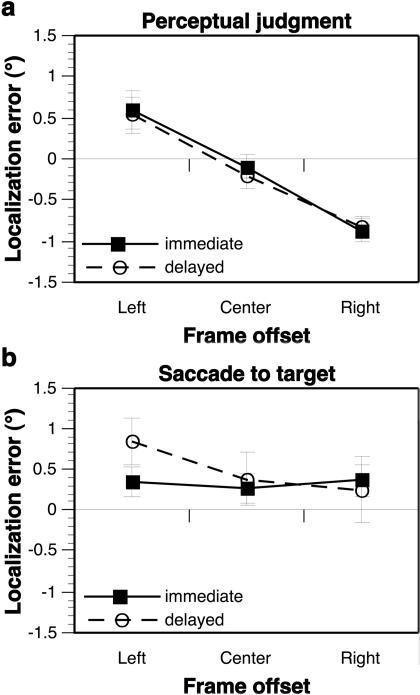
Perceptual and Sensorimotor Roelofs Effects (A) Effect of frame location on immediate (solid line) and delayed (dashed line) perceptual judgments of target location, with a significant main effect of frame offset but no frame × delay interaction (Table 1); error bars represent the standard error of the mean localization errors for each subject. (See also Figure S1A for a time line of the task events, Figure S1B for a plot of the Roelofs effect for each of the individual target locations, and Figure S1C for plots of the Roelofs effect within individual subjects.) (B) Effect of frame offset on immediate (solid line) and delayed (dashed line) saccadic eye movements, with a significant main effect of frame offset and a significant frame × delay interaction. When tested separately, the main effect of frame offset was not significant for immediate responses, but was significant for delayed responses (Table 1; see also Figure S2).

We first sought to replicate the findings of Bridgeman et al. (1997) by testing subjects' abilities to indicate the locations of targets presented within the context of a centered frame or one displaced 5° left or right of the midline. Subjects provided a perceptual report of each target location by comparing it to an array of five possible target locations (−4°, −2°, 0°, 2°, and 4° from the subject's midline, at eye level) learned during an earlier training session. As had been demonstrated previously (Bridgeman et al. 1997), the displaced frame did cause a mislocalization of the target, whether the subject responded immediately after the offset of the target and frame (Figure 2A, solid line; Table 1) or after a 4-s delay period during which the subject was in complete darkness (Figure 2A, dashed line; Table 1). The size of this illusion was quantified by subtracting the magnitude of the localization bias caused by a right-shifted frame from that caused by a left-shifted frame, resulting in an effect size of 1.47° ± 0.32° (mean ± SEM) across all subjects for immediate responses and 1.37° ± 0.30° for delayed responses.

**Table 1:**
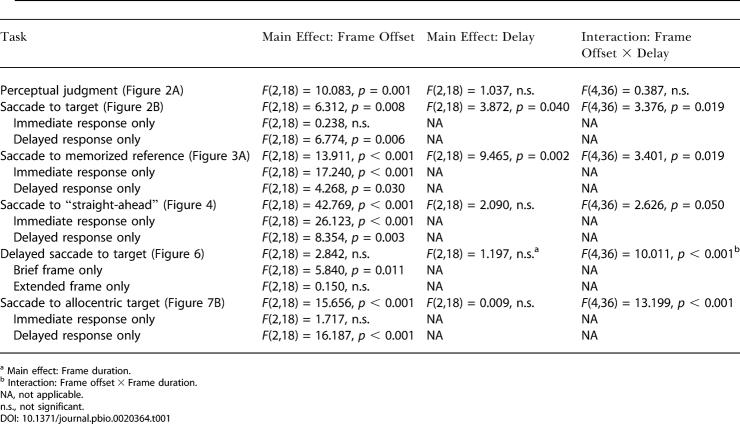
Significance of Effects

^a^ Main effect: Frame duration

^b^ Interaction: Frame offset × Frame duration

NA, not applicable

n.s., not significant

A second group of subjects was instructed to make open-loop saccadic eye movements to the target location. Saccades performed immediately after the frame and target were extinguished showed no significant effect of frame position (effect size = −0.01° ± 0.13°, Figure 2B, solid line; Table 1). This finding replicated the general pattern of sensorimotor responses described by Bridgeman et al. (1997) and extended them by demonstrating that immediate saccadic eye movements, like pointing movements of the hand, can be guided accurately to targets that are perceptually mislocalized. However, when subjects were required to withhold this sensorimotor response during a 4-s delay period, the eventual saccadic eye movement did reflect a small but significant Roelofs effect (effect size = 0.60° ± 0.26°, Figure 2B, dashed line; Table 1). Again, this delayed sensorimotor Roelofs effect replicated the findings of Bridgeman et al. (1997).

### A Mislocalization of the Target or of the Comparison Array?

**Figure 3:**
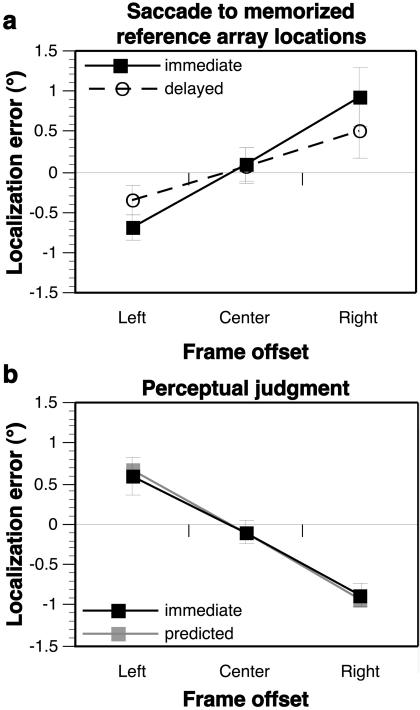
Inverse Roelofs Effect for Remembered Space (A) An inverse Roelofs effect for immediate (solid line) and delayed (dashed line) sensorimotor responses toward remembered reference array locations, with a significant main effect of frame offset and a significant frame × delay interaction. When tested separately, the main effect of frame offset was significant for both immediate and delayed responses (Table 1; see also Figure S3). (B) The inverse Roelofs effect for remembered space can be used to predict the pattern of the Roelofs effect for targets presented within an offset frame. For example, a frame offset to the right would cause the remembered comparison array to be mislocalized as being shifted approximately 1° to the right (from Figure 3A, solid line); a target presented at the center location of the comparison array (i.e., at the objective midline) would therefore be reported to lie approximately 1° to the left of the remembered center location. Computed in this way for all target and frame locations, the predicted Roelofs effect (gray lines and data points) closely matched the measured Roelofs effect for the perceptual judgment (black lines and data points, from Figure 2A, solid line), with a predicted effect size (1.61°) that did not significantly differ from the measured effect (1.47° ± 0.32°; t[9] = 0.44, n.s.). Furthermore, the measured Roelofs effect did not differ from the predicted effect for any individual frame position (left frame: t[9] = 0.39, n.s.; center frame: t[9] = 0.01, n.s.; right frame: t[9] = 0.36, n.s.).

To test the hypothesis that the perceptual Roelofs effect can be explained by a memory of the comparison array that is shifted in the direction of the frame, subjects were asked to indicate the remembered locations of the five items within the array of possible targets that was learned in complete darkness during the earlier training session. In experimental trials, a centered or offset frame was presented near the time that an auditory cue instructed the subject to make a saccadic eye movement to one of the remembered locations. If an offset frame caused a distortion in the memory of the reference array, the accuracy of the saccadic responses would be affected. This was indeed the case, with targets mislocalized in the same direction as the displacement of the offset frame (effect size = −1.61° ± 0.32°, Figure 3A, solid line; Table 1). Since this pattern of mislocalization for remembered targets was in the opposite direction of the normal Roelofs effect reported by Bridgeman et al. (1997) and replicated above (see Figure 2A), we refer to it as an *inverse Roelofs effect for remembered space.* This finding provides strong evidence that the perceptual errors associated with the normal Roelofs effect are most parsimoniously explained by the subject's comparison of the target location with a distorted memory of the comparison array. As a further test of this hypothesis, it is possible to use the pattern of mislocalizations evident with the inverse Roelofs effect for remembered space to predict a subject's perceptual report when comparing a target location with the inaccurately remembered comparison array. The resultant prediction for the perceptual Roelofs effect very closely matched the measured Roelofs effect (see Figure 3B), with a predicted effect size (1.61°) that did not significantly differ from the measured effect size (1.47° ± 0.32°). Thus, the inverse Roelofs effect for remembered space effectively accounts for the mislocalizations that occurred when subjects provided perceptual reports of the locations of targets presented within the context of an offset frame.

### Distortion of the Apparent Midline

**Figure 4:**
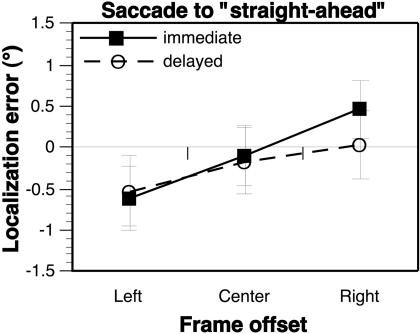
Inverse Roelofs Effect for the Apparent Midline An inverse Roelofs effect for immediate (solid line) and delayed (dashed line) sensorimotor responses toward the apparent midline, with a significant main effect of frame offset and a significant frame × delay interaction. When tested separately, the main effect of frame offset was significant for both immediate and delayed responses (Table 1; see also Figure S4).

Although the inverse Roelofs effect for remembered space provides an explanation for the perceptual mislocalization that occurs in the presence of an offset frame, the mechanism whereby the offset frame is capable of distorting remembered space remains to be explained. The locations of the items within the comparison array were learned in complete darkness and therefore could only be localized in egocentric coordinates, perhaps with respect to the subject's apparent midline (Mergner et al. 2001). Under normal conditions, the center of the visual field would serve as an accurate indicator of straight-ahead. However, the impoverished visual environment of the present experiment contained only the large rectangular frame, which might have served to attract the apparent midline in the direction of the frame's offset (Werner et al. 1953; Brosgole 1968; Brecher et al. 1972; Dassonville et al. 2004), dragging the spatial memory of the comparison array with it. To directly test the hypothesis that the offset frame in the current context is capable of biasing the apparent midline, subjects were asked to perform a version of the task in which they were simply asked to “look straight ahead” immediately after the presentation of a centered or offset frame. Subjects' reports of “straight-ahead” were indeed found to be affected by the presence of the frame, with the movements biased in the same direction as the offset frame (effect size = −1.08° ± 0.14°, with a negative value once again reflecting an effect in the direction opposite the normal induced Roelofs effect; Figure 4, solid line; Table 1).

**Figure 5:**
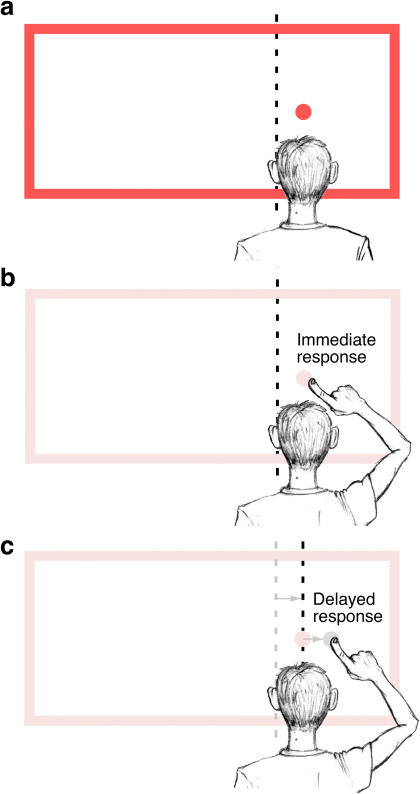
The Biased-Midline Hypothesis (A) A depiction of the manner in which a target (red circle), located directly in front of the subject, would be perceived as being a small distance to the right of the subject's apparent midline (dotted line), which has itself been biased to the left in the presence of the left-shifted frame. (B) An immediate open-loop sensorimotor response (pointing movement, as shown here, or saccade begun immediately after the target and frame are extinguished) would be accurate if the goal of the movement were encoded in the same distorted reference frame (that is, a small distance to the right of the distorted apparent midline). (C) With the frame and target extinguished during an imposed delay, the apparent midline would drift back to veridical (gray arrows), dragging the remembered location of the target (gray circle) with it. A subsequent sensorimotor response aimed at the remembered target (located a small distance to the right of the now-veridical apparent midline) would result in a delayed sensorimotor Roelofs effect.

These findings can also explain the absence of errors seen with immediate sensorimotor responses, if one assumes that the movements are guided within the same distorted frame of reference that is used to encode the target location. For example, a target presented at the subject's true midline in the presence of a left-shifted frame would be encoded by the brain as having been located a small distance to the right of the apparent midline (which itself has been pulled leftward by the frame; Figure 5A). If the corresponding sensorimotor response is guided within this same distorted reference frame, the eye or hand would be expected to move to a location just to the right of the distorted apparent midline (Figure 5B). In essence, the error in target localization would be exactly cancelled by the error in motor guidance, resulting in an accurate response. Thus, an accurate sensorimotor response is fully expected when the target and response are encoded within the same distorted map of space.

### Transient Effects of the Illusion

In contrast to the stable Roelofs effect with delayed perceptual responses (see Figure 2A, dashed line), we found that the inverse Roelofs effect for remembered space was diminished (to an effect size = −0.87° ± 0.28°) when a 4-s delay was imposed between the frame presentation and the saccade to a remembered item in the comparison array (see Figure 3A, dashed line). Similarly, the effects of an offset frame on the apparent midline diminished during a delay imposed after the frame was extinguished (to an effect size = −0.56° ± 0.15°; see Figure 4, dashed line). These findings demonstrate that the distortions of the apparent midline and remembered space are transient, decreasing over time when the offset frame is no longer visible. However, even after a delay of 4 s, responses were still somewhat biased by the preceding frame, indicating either an extended time course during which the effects of the frame dissipate or a hysteresis that prevents the apparent midline from becoming fully veridical in the absence of visual input.

The transient nature of the apparent midline distortion can also provide an explanation for the increase in Roelofs effect seen when a delayed saccade is made to a target presented within the offset frame (see Figure 2B, dashed line). As an example, let us once again assume a target presented at the subject's true midline, in the presence of a left-shifted frame. During the imposed delay, it is reasonable to assume that the memory of the target's location would be encoded with respect to the distorted apparent midline (Mergner et al. 2001)—in our example, the target would be remembered as being a small distance to the right of the apparent midline, which has been pulled leftward by the frame (see Figure 5A). After the frame is removed and its distorting influences diminish, the apparent midline would drift back toward its veridical orientation under the influence of vestibular (Fischer and Kornmueller 1930; Morant 1959) and proprioceptive (Karnath 1999) inputs, dragging the remembered target location with it. The delayed response would then be directed to this incorrectly remembered location, just to the right of the newly corrected apparent midline (see Figure 5C). If this account were true, one would expect the normal Roelofs effect for sensorimotor responses to increase during a delay by an amount comparable to the decrease in the inverse Roelofs effect for delayed movements directed to the apparent midline or items in the remembered comparison array. Indeed, the current studies found the sensorimotor Roelofs effect to increase 0.61° during the imposed delay (that is, from −0.01° to 0.60°; see Figure 2B, solid versus dashed lines), while the inverse Roelofs effect decreased 0.74° for movements to items in the comparison array (see Figure 3A, solid versus dashed lines) or 0.52° for movements to indicate the apparent midline (see Figure 4, solid versus dashed lines). In contrast, one would not expect an imposed delay to have any effect on a perceptual report of target location, since the relative relationship of the remembered target and the remembered comparison array would remain unchanged as the apparent midline returned to veridical.

**Figure 6:**
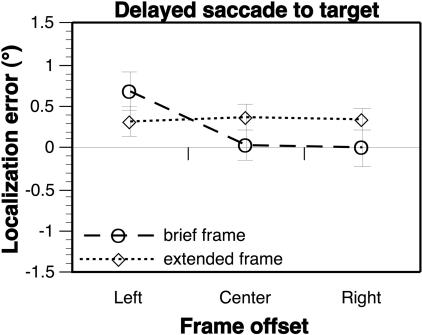
Effect of the Frame during the Delay Period Effect of frame offset on delayed saccadic eye movements, for trials in which the frame was either extinguished at the start of the delay period (brief frame, dashed line), or was present throughout the delay (extended frame, dotted line). There was a significant frame × delay interaction; when tested separately, the main effect of frame offset was not significant for the extended frame duration, but was significant for the brief duration, replicating the results shown in Figure 2B, dashed line (Table 1; see also Figure S5).

To more closely examine the hypothesis that the increase in the Roelofs effect for delayed sensorimotor responses is due to a drift of the apparent midline back to veridical after the frame is extinguished, a group of subjects performed a version of the delayed saccade task in an experiment in which, on half of the trials, the frame continued to be visible during the 4-s delay period, disappearing only when the subject received the verbal cue to respond. In the other half of the trials, the frame and target were extinguished simultaneously, with the subject sitting in complete darkness during the delay. When the frame was absent during the delay, the eventual response was significantly affected by frame position (Figure 6, dashed line; Table 1), replicating the results from our previous delayed saccade task (see Figure 2B, dashed line). In contrast, for those trials in which the frame was present during the delay, no effect of frame position was evident (see Figure 6, dotted line; Table 1). Thus, it seems that the continued presence of the frame maintains the apparent midline in a biased orientation, such that the errors in target encoding are cancelled by the errors of motor guidance even after a delay. Since the transient nature of the Roelofs effect is specifically not a function of the delay from target presentation to response (but rather depends on the delay from frame offset), these findings argue against the hypothesis of Bridgeman et al. (1997) that the transience reflects a lack of memory for target location within a system that guides the sensorimotor responses.

### Reevaluating the Need for Separate Perception and Action Systems

**Figure 7:**
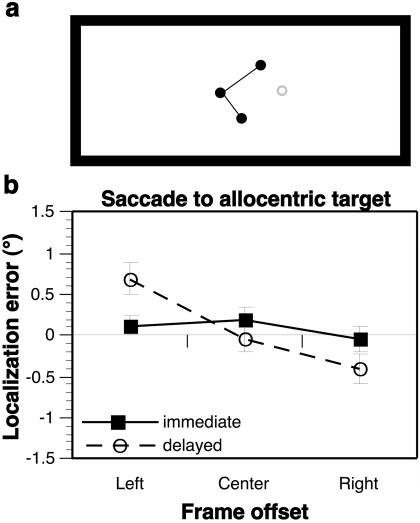
A Roelofs Effect for Allocentrically Defined Targets (A) Visual display used to define allocentric targets; subjects were instructed to move the eyes to the missing corner of the partial rectangle (gray circle, not seen by subject). During experimental trials, this stimulus array was presented within a large rectangular frame that was either centered or offset left or right of the subject's midline. (B) Effect of frame offset on immediate (solid line) and delayed (dashed line) sensorimotor responses to targets defined allocentrically, with a significant main effect of frame offset and a significant frame × delay interaction. When tested separately, the main effect of frame offset was not significant for immediate responses, but was significant for delayed responses (Table 1; see also Figure S6)

While the present findings provide an alternative explanation for the behavioral dissociation of Roelofs illusion, it could still be argued that they do not completely rule out the possibility of separate systems for perception and action. For example, it could be that there does exist a context-independent “action” system whose only function is to guide movements aimed immediately and directly toward a currently visible target, and a “perceptual” system capable of guiding all other movements (e.g., movements to remembered targets, to mirror-image locations of currently visible targets [Dassonville et al. 2004], or to indicate straight-ahead, all of which reflect the errors associated with Roelofs effect). If this were true, then it would be useful to contrast the capabilities of the action system with those of the perceptual system under equivalent conditions (i.e., for movements guided immediately and directly to currently defined targets). To do this, we designed an experiment in which subjects were asked to make saccadic eye movements to targets that were defined purely through the use of contextual cues that could serve as targets only for the presumed perceptual system, if the action system truly operates in a context-independent fashion. Specifically, stimuli consisted of three corners (and two sides) of a rectangle, with the target location defined as the missing corner (Figure 7A). These stimuli were then presented within a large rectangular frame that was centered or offset from the subject's midline. The pattern of mislocalizations seen with these allocentrically defined targets was identical to that seen with real targets (compare Figure 7B to Figure 2B), with no Roelofs effect evident for immediate responses (Figure 7B, solid line; Table 1), but a significant effect evident for responses delayed by 4 s (Figure 7B, dashed line; Table 1). Thus, if separate action and perception systems do exist, it would seem that they are not differently abled with regard to Roelofs effect after all. Instead, both would be capable of guiding immediate movements accurately in spite of the Roelofs effect, with the action system simply immune to the Roelofs distortions, while the perceptual system would be required to guide movements within the same distorted reference frame as the target is encoded, so that the errors cancel, as described above. While it is technically possible that the brain would maintain two such redundant systems for guiding movements, it seems improbable. Instead, a more parsimonious explanation for the behavioral dissociation that accompanies Roelofs effect is provided by the brain's use of a single reference frame whose origin (the apparent midline) is transiently distorted by the presence of an offset frame for both perceptual judgments and sensorimotor responses.

### Further Evidence for the Use of Contextual Information in Motor Control

As is the case for many illusions, the distortion of visual space associated with Roelofs illusion would seem to be a by-product of the brain's use of contextual cues that—under normal circumstances—would provide additional information that could allow for the creation of a more accurate neural representation of the world. After all, most perceptual judgments and movements are made within the context of a well-lit, highly structured visual scene, the center of which would normally provide an accurate indicator of straight-ahead. Given this, it would be somewhat surprising if the beneficial information that is provided by contextual cues under normal circumstances were used for perception but not for motor control. Indeed, many previous investigations have demonstrated that contextual cues do affect the guidance of movements, even those directed to currently visible targets. For example, the accuracy and kinematics of open-loop pointing movements are greatly affected by the presence of a small distractor (Howard and Tipper 1997; Tipper et al. 1997; Gangitano et al. 1998) or a well-lit, highly structured visual scene (Foley 1975; Conti and Beaubaton 1980; Blouin et al. 1993; Toni et al. 1996). This has also been demonstrated using paradigms in which the visual representation of target location is first distorted by altering the relationship between actual eye position and the brain's representation of eye position (e.g., by paralyzing the extraocular muscles with curare [Matin et al. 1982], stretching them [Stark and Bridgeman 1983], fatiguing them [Shebilske 1984], or vibrating them [Velay et al. 1994]). Although these distortions of represented eye position have been shown to cause errors in open-loop pointing movements aimed at targets presented in otherwise complete darkness, the presence of a highly structured visual scene significantly reduces the magnitude of these errors. Contextual cues are also used by the oculomotor system to minimize the errors of saccadic eye movements directed toward targets presented near the time of a preceding saccade (Honda 1993, 1999; Dassonville et al. 1995). Furthermore, several other studies have clearly demonstrated that illusion-causing contextual cues can affect the dynamic characteristics of pointing and grasping movements (Smeets and Brenner 1995; Brenner and Smeets 1996; Gentilucci et al. 1997; van Donkelaar 1999; Jackson and Shaw 2000; Westwood et al. 2001; Bartelt and Darling 2002) and the accuracy of eye movements (Festinger et al. 1968; Binsted and Elliott 1999; Both et al. 2003; McCarley et al. 2003; Sheliga and Miles 2003).

### Additional Evidence against a Simple Perception/Action Dissociation

Although previous authors have suggested that the perceptual effects of Roelofs illusion could be explained by a distortion of the apparent midline and egocentric reference frame (Werner et al. 1953; Brosgole 1968; Brecher et al. 1972; Dassonville and Bala 2004; Dassonville et al. 2004), we have demonstrated here that this same transient distortion can also provide a full, precise, and mechanistic explanation of the immediate and delayed sensorimotor effects of Roelofs illusion. By extending this hypothesis to include dynamic visual displays, a similar mechanism can also be used to explain the behavioral dissociation seen with illusions of induced motion (Bridgeman et al. 1981; Wong and Mack 1981). Most important, this hypothesis accounts for both phenomena without relying on an assumption of separate neural maps of space for perception and action. Given this, the behavioral dissociation evident with these illusions cannot be used as evidence that exclusively supports the existence of a perception/action dissociation in visual processing.

Of course, several other behavioral studies can still be pointed to as evidence for a perception/action dissociation in visual processing. However, many of these studies have recently come under intense scrutiny, with some researchers failing to replicate previously reported dissociations once important control conditions were included (Honda 1990; Dassonville et al. 1992; Pavani et al. 1999; Franz et al. 2000; Franz 2003). Other researchers have proposed alternative explanations for obvious behavioral dissociations by pointing out that the perception and action tasks differed along other dimensions as well (e.g., semantic versus pragmatic requirements [Jeannerod 1997], relative versus absolute judgments [Vishton et al. 1999], allocentric versus egocentric reference frames [Bruno 2001], and size versus position judgments [Smeets and Brenner 2001]). Similarly, in studies that have purported to demonstrate a perception/action dissociation in patients with dorsal and ventral lesions, it can be argued that the behavioral tests used to characterize the deficits also suffered from these same confounds. For example, Dijkerman et al. (1998) found that a patient (DF) with a ventral lesion was impaired in a task that required her to reach for and grasp an object by placing her fingers in two or three circular holes whose locations were varied from trial to trial. Although these findings led Dijkerman et al. (1998) to conclude that movements like these must be controlled by a ventral perceptual system that happens to operate within an allocentric reference frame, it is also possible to interpret these data as suggesting that the lesion simply caused a specific deficit in allocentric encoding rather than a general deficit in perception. This same patient has also been found to be impaired in perceiving the visual pitch of a plane tilted from vertical, even though that same plane causes a distortion of her perception of vertical eye level, just as it does in healthy subjects (Servos et al. 1995). Thus, although DF demonstrates a dissociation in her ability to use information concerning visual pitch, it is a dissociation of two perceptual measures and specifically *not* a dissociation of perception and action.

While it seems clear that there do exist at least some examples of dissociations in the accuracy of various behavioral responses from normal subjects and patients, a great deal of evidence now suggests that these dissociations cannot simply be attributed to separate systems for perception and action. Indeed, the dorsal and ventral processing streams are both composed of a myriad of functionally distinct and highly interconnected visual processing areas, each with its own mechanisms for representing various aspects of the visual world. Each visuomotor task would undoubtedly rely on the processing capabilities of a subset of these areas, with different tasks relying on different subsets depending on their precise requirements. With this in mind, it seems overly simplistic to consider visuomotor behavior as being derived from the function of only one of two distinct processing streams. Rather, it is more plausible that flexible functional networks would form among the areas required to play a role in the completion of the task at hand. The characteristics of the behavioral performance would then be dependent on the representational idiosyncrasies of those areas involved (McGraw et al. 2003).

Several previous studies of the perceptual and motor effects associated with various visual illusions have indicated an apparent dissociation of visual pathways for perception and action, with perception generally found to be prone to illusions to which actions are immune. These conclusions, however, are not without controversy; as described above, several other studies have questioned the perception/action dissociation attributed to many illusions, after contrary evidence or alternative explanations were produced. A notable exception to this has been the induced Roelofs effect, where the presumption of a perception/action dissociation has remained unquestioned since it was originally proposed (Bridgeman et al. 1997). The results presented here, however, point to a brain mechanism for spatial localization that can fully and precisely explain the behavioral dissociation of the induced Roelofs effect without requiring the existence of separate neural systems for perception and action. The visual image of a large frame, whose center is offset left or right of an observer's midline, was demonstrated to cause a transient distortion of an observer's egocentric reference frame by biasing the apparent midline. Within this distorted reference frame, objects are perceived to be located in a direction shifted opposite that of the frame offset. The fact that movements of the eyes and hands can be accurately directed to this misperceived target location can be explained by a cancellation of errors that occurs when the movement is guided within the same distorted reference frame. Thus, these findings indicate that both perceptual judgments and motor responses are based upon either a single map of space or separate maps that are equally prone to the distortion caused by the Roelofs effect.

## Materials and Methods

### 

#### Subjects

In each experiment, ten subjects (undergraduate students of the University of Oregon) provided informed consent to participate and were compensated with either course credit or a small monetary payment, as per a protocol approved by the University of Oregon Committee for the Protection of Human Subjects/Institutional Review Board.

#### Visual display

Subjects were placed in a completely darkened room and presented with a visual display that was back-projected (Cine7 projector, Barco, Kuurne, Belgium) onto a screen measuring 128 × 96 cm, positioned 122 cm from the eyes. Visual targets were small (0.35° of visual angle, 100-ms duration) red spots, located −4°, −2°, 0°, 2°, or 4° from the subject's midline, at eye level. During experimental trials, targets were presented within a large red unfilled frame (21° horizontal × 8.5° vertical, 1° thickness; 1,000-ms duration) that was either centered with respect to the subject's midline or shifted 5° left or right of the midline. All visual images were presented on a black background, with the high contrast of the Barco Cine7 projector preventing subjects from seeing the edges of the screen.

#### Eye movement monitoring

Head and binocular eye positions were monitored at 250 Hz with an eye tracker (Eyelink; SensoMotoric Instruments, Needham, Massachusetts, United States) that allowed head-free measurement of gaze (precision = 0.01°); however, target placement was such that head movements contributed to only a small fraction of the total gaze displacement on any trial. To start each session (and as necessary throughout each session), eye-tracker calibration was performed using a 3 × 3 grid of targets spaced 13.5° apart in the horizontal dimension and 10.5° apart in the vertical dimension. For each subject, the average fixation error across the nine calibration targets was required to be 1° or less before beginning the subsequent practice and experimental trials; thus, absolute tracking errors within this calibration field were at most 1° in magnitude and were typically only 0.5°. In addition, subjects began each experimental trial by directing the eyes to a fixation point and pressing the space bar of a keyboard to indicate readiness. Upon this signal, the eye-tracker computer performed an adjustment of the calibration to correct for any drift that had occurred since the onset of the previous trial; that is, the calibration was adjusted so that the signal of eye position matched the known location of the fixation point. In experiments requiring eye movement responses, the gaze signals from the two eyes were averaged to yield a single representation of gaze direction as the dependent variable.

#### Behavioral tasks

Each experiment was preceded by a set of practice trials (36–71 trials) in which subjects performed the appropriate task (see below) in the absence of the large Roelofs-inducing rectangular frame. Feedback was provided at the end of each practice trial to assist subjects in improving their performance. For all experiments, feedback included the illumination of a small circle at the target location. For the experiment in which subjects provided a button press to report the perceived target identity, a computer-generated voice also provided auditory feedback to indicate the correct target identity. For all experiments in which subjects indicated the target position with a saccadic eye movement, feedback following each practice trial also included the illumination of a small square to indicate the final gaze position; subjects were instructed to use the feedback in an attempt to minimize the distance between the target and final gaze positions. No feedback concerning response accuracy was ever provided during the experimental trials.

To test the perceptual effects of Roelofs illusion (see Figure 2A; see also Figure S1A), subjects were first trained to recognize targets presented in each of the five possible target locations. Each trial began with a fixation point (centered horizontally, 8.5° above eye level) that was extinguished 1150–1650 ms before the onset of the target (100-ms duration). A computer-generated voice (“Respond,” presented just before or 4 s after the target) provided subjects with a temporal cue to press one of five keys on the computer keyboard to indicate the identity of the target based on its perceived location (using the right hand, thumb = extreme left target, little finger = extreme right target, etc.). Throughout each trial, subjects were required to maintain gaze within an invisible window of 4° centered on the fixation point, even after the fixation point was removed. At the end of each practice trial, the target was displayed again to provide visual feedback, and the computer-generated voice provided a verbal indication of the target's actual location. Subsequent experimental trials had a similar time course, except for the inclusion of a large frame (1,000-ms duration) that was illuminated 900 ms before target onset (frame and target were extinguished simultaneously). Subjects in this and all other tasks were explicitly instructed to ignore the presence of the large frame when making their judgments of target location.

Similar trials were used to test the sensorimotor effects of Roelofs illusion (see Figure 2B; see also Figure S2A), with the exception that subjects were instructed to make a saccadic eye movement to the target location after being cued to “Respond” by the computer-generated voice. After making any necessary eye movements to fixate the remembered target location, subjects ended each trial by pressing the “Enter” key on the keyboard (this final gaze position was used as the subject's indication of target location). Subjects in this version of the task were never informed that there were only five possible target locations.

To test the effects of Roelofs illusion on remembered visual space (see Figure 3A; see also Figure S3A), subjects were instructed to make eye movements to the remembered locations of the five possible targets. During practice trials in which no frames were presented, the visual target was replaced with a computer-generated voice providing the identity of the target location (“One” = extreme left target, “Five” = extreme right target, etc.). After the computer-generated cue to “Respond” (presented just before or 4 s after the cue for target identity), subjects moved their eyes to the remembered location of the target and ended the trial by pressing the “Enter” key. Feedback during practice trials was provided in the way of an illumination of the correct target location and a small square indicating the final gaze position for that trial. In subsequent experimental trials, the large rectangular frame was presented 900 ms before the onset of the auditory target-identity cue, and no feedback was provided.

To test the effects of Roelofs illusion on the apparent midline (see Figure 4; see also Figure S4A), subjects were instructed to make eye movements to look straight-ahead when cued to “Respond” by the computer-generated voice. To prevent the fixation point from providing information about the actual midline in this version of the task, the horizontal position of the fixation point was varied randomly from trial to trial (−3°, −1°, 1°, or 3° from midline).

To determine whether the delayed sensorimotor Roelofs effect was modulated by the presence of the frame during the imposed delay period (see Figure 6; see also Figure S5A), all trials contained an imposed delay of 4 s between target presentation and the subsequent computer-generated “Respond” command. In half of the trials, the rectangular frame (1-s duration) was extinguished simultaneous with the target (these trials exactly replicated those used to originally measure the delayed sensorimotor Roelofs effect; see Figure 2B, dashed line). In the remaining trials, the frame (5-s duration) remained illuminated until the onset of the computer-generated “Respond” command.

To test the effects of Roelofs illusion on saccades directed toward targets defined allocentrically (see Figure 7; see also Figure S6A), stimuli consisted of three small circles (connected by two thin lines), indicating three of the four corners of a small rectangle whose size and orientation was varied randomly from trial to trial. The partial rectangle was positioned so that the missing corner was located −4°, −2°, 0°, 2°, or 4° from the subject's midline, at eye level. Subjects were instructed to move their eyes to the location of the missing corner when cued to “Respond.” To accommodate the larger space required to define the target location allocentrically, the Roelofs-inducing frame was enlarged (28° horizontal × 14° vertical, 1° thickness) in this version of the experiment.

#### Statistical analysis

In each experiment, the signed magnitudes of localization errors (i.e., the difference between the actual and reported locations of the target in the horizontal dimension, with six repetitions for each trial type) were analyzed with a full-factorial analysis of variance, in a 5 (target location) × 3 (frame position) × 2 (response delay) design (see Figures 2, 3, and 7), a 5 (target location) × 3 (frame position) × 2 (frame duration) design (see Figure 6), or a 4 (fixation location) × 3 (frame position) × 2 (response delay) design (see Figure 4). In the present analyses, only the effects of frame position, frame duration, and response delay are considered. The effect of target location has been explored elsewhere (Dassonville and Bala 2004).

## Supporting Information

Figure S1 Time Line and Results for the Perceptual Roelofs Effect (A) Time line of task events for immediate (black) and delayed (gray) perceptual judgments of target location. Note that in this and all other experiments, feedback was presented only during practice trials, and the frame was presented only during experimental trials.
(B) Effect of frame location on immediate (solid line) and delayed (dashed line) perceptual judgments of target location for each of five target locations.
(C) Effect of frame location on immediate (solid line) and delayed (dashed line) perceptual judgments of target location for each of ten subjects.
(579 KB TIF).

Figure S2 Time Line and Results for the Sensorimotor Roelofs Effect (A) Time line of task events for immediate (black) and delayed (gray) sensorimotor responses.
(B) Effect of frame offset on immediate (solid line) and delayed (dashed line) sensorimotor responses for each of five target locations.
(C) Effect of frame offset on immediate (solid line) and delayed (dashed line) sensorimotor responses for each of ten subjects.
(1.1 MB TIF).

Figure S3 Time Line and Results for the Inverse Roelofs Effect on Remembered Space (A) Time line of task events for immediate (black) and delayed (gray) sensorimotor responses toward remembered reference-array locations.
(B) An inverse Roelofs effect for immediate (solid line) and delayed (dashed line) sensorimotor responses toward remembered reference-array locations, for each of five target locations. The nonlinear effect of target location has been addressed elsewhere (Dassonville and Bala 2004).
(C) An inverse Roelofs effect for immediate (solid line) and delayed (dashed line) sensorimotor responses toward remembered reference array locations, for each of ten subjects.
(2.5 MB TIF).

Figure S4 Time Line and Results for the Inverse Roelofs Effect on the Apparent Midline (A) Time line of task events for immediate (black) and delayed (gray) sensorimotor responses toward the apparent midline.
(B) An inverse Roelofs effect for immediate (solid line) and delayed (dashed line) sensorimotor responses toward the apparent midline, for each of ten subjects.
(856 KB TIF).

Figure S5 Time Line and Results for Testing the Effects of Frame Duration (A) Time line of task events for delayed sensorimotor responses, for trials in which the frame was either extinguished at the start of the delay period (brief frame, black) or was present throughout the delay (extended frame, gray).
(B) Effect of frame offset on delayed sensorimotor responses, for trials in which the frame was either extinguished at the start of the delay period (brief frame, dashed line), or was present throughout the delay (extended frame, dotted line), for each of five target locations.
(C) Effect of frame offset on delayed sensorimotor responses, for trials in which the frame was either extinguished at the start of the delay period (brief frame, dashed line) or was present throughout the delay (extended frame, dotted line), for each of ten subjects.
(4.7 MB TIF).

Figure S6 Time Line and Results for the Roelofs Effect on Allocentrically Defined Targets (A) Time line of task events for immediate (black) and delayed (gray) sensorimotor responses to targets defined allocentrically.
(B) Effect of frame offset on immediate (solid line) and delayed (dashed line) sensorimotor responses to targets defined allocentrically, for each of five target locations.
(C) Effect of frame offset on immediate (solid line) and delayed (dashed line) sensorimotor responses to targets defined allocentrically, for each of ten subjects.
(1.8 MB TIF).
