# Supplementary figures and images for: Perception, Action, and Roelofs Effect: A Mere Illusion of Dissociation
Source: PLoS Biol. 2004 Oct 26;2(11):e364. doi: 10.1371/journal.pbio.0020364 (PMC524248; doi:10.1371/journal.pbio.0020364)

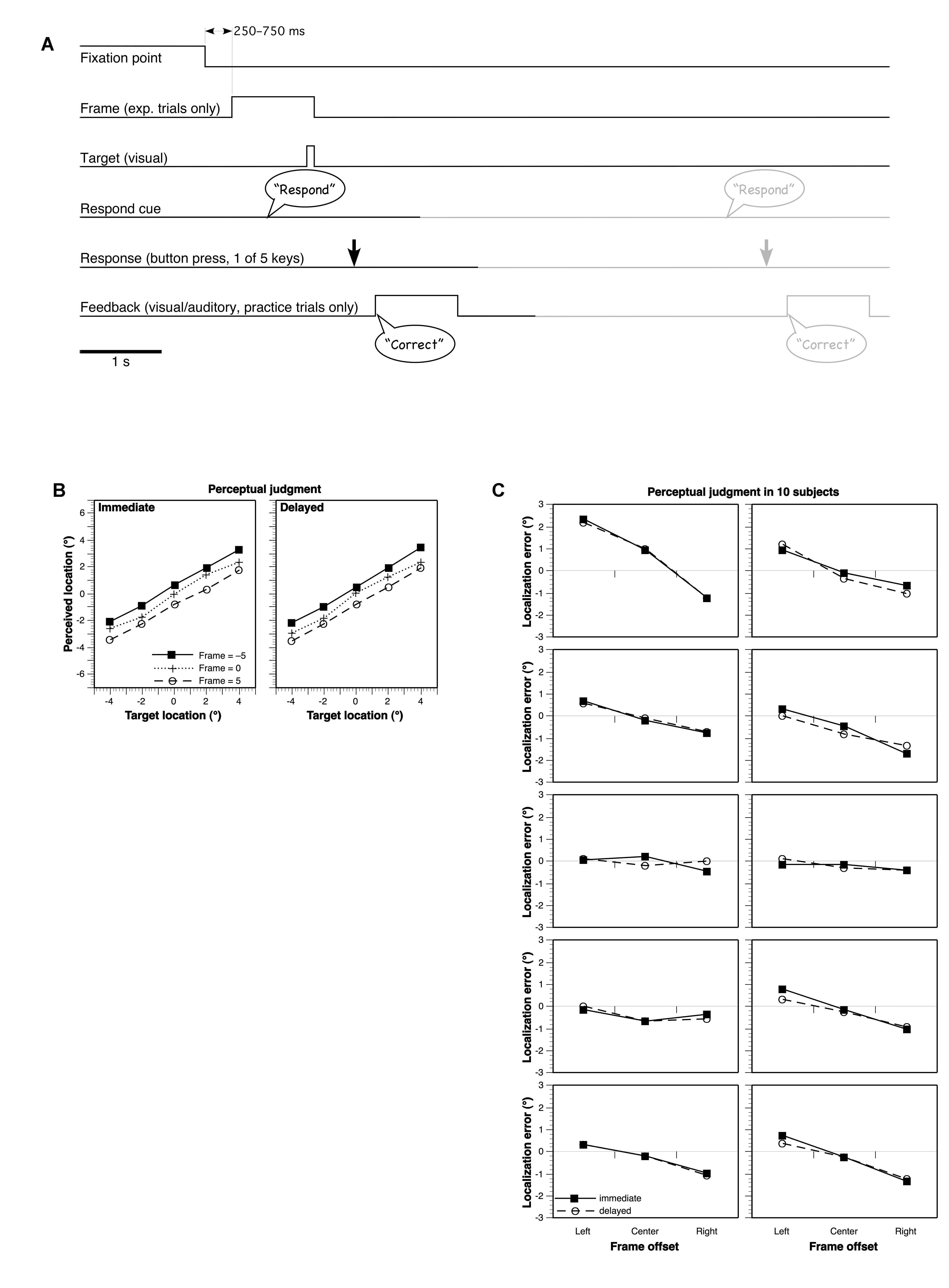

Supplement: Figure S1 — (A) Time line of task events for immediate (black) and delayed (gray) perceptual judgments of target location. Note that in this and all other experiments, feedback was presented only during practice trials, and the frame was presented only during experimental trials. (B) Effect of frame location on immediate (solid line) and delayed (dashed line) perceptual judgments of target location for each of five target locations. (C) Effect of frame location on immediate (solid line) and delayed (dashed line) perceptual judgments of target location for each of ten subjects. (579 KB TIF). [file pbio.0020364.sg001.tif]

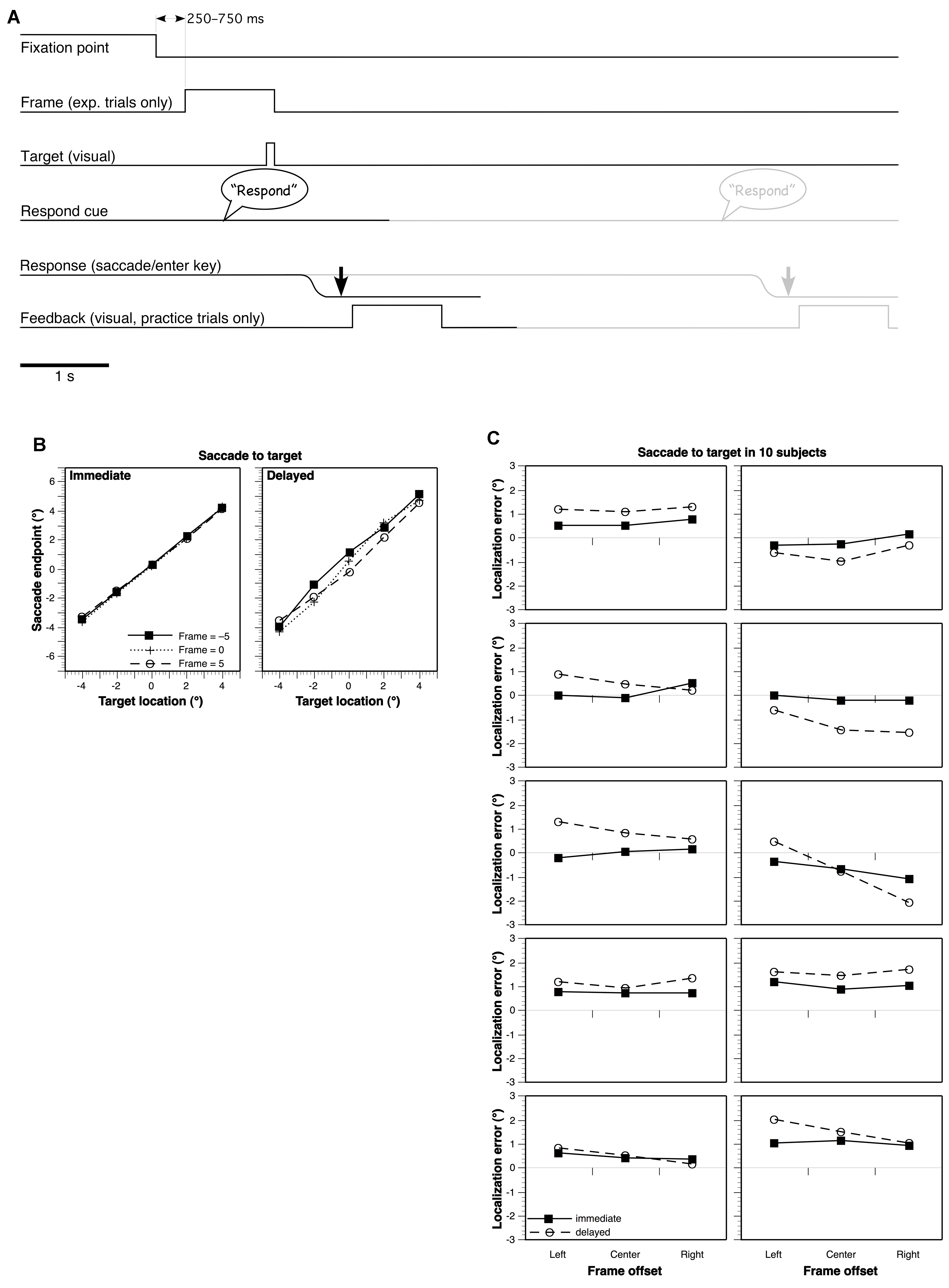

Supplement: Figure S2 — (A) Time line of task events for immediate (black) and delayed (gray) sensorimotor responses. (B) Effect of frame offset on immediate (solid line) and delayed (dashed line) sensorimotor responses for each of five target locations. (C) Effect of frame offset on immediate (solid line) and delayed (dashed line) sensorimotor responses for each of ten subjects. (1.1 MB TIF). [file pbio.0020364.sg002.tif]

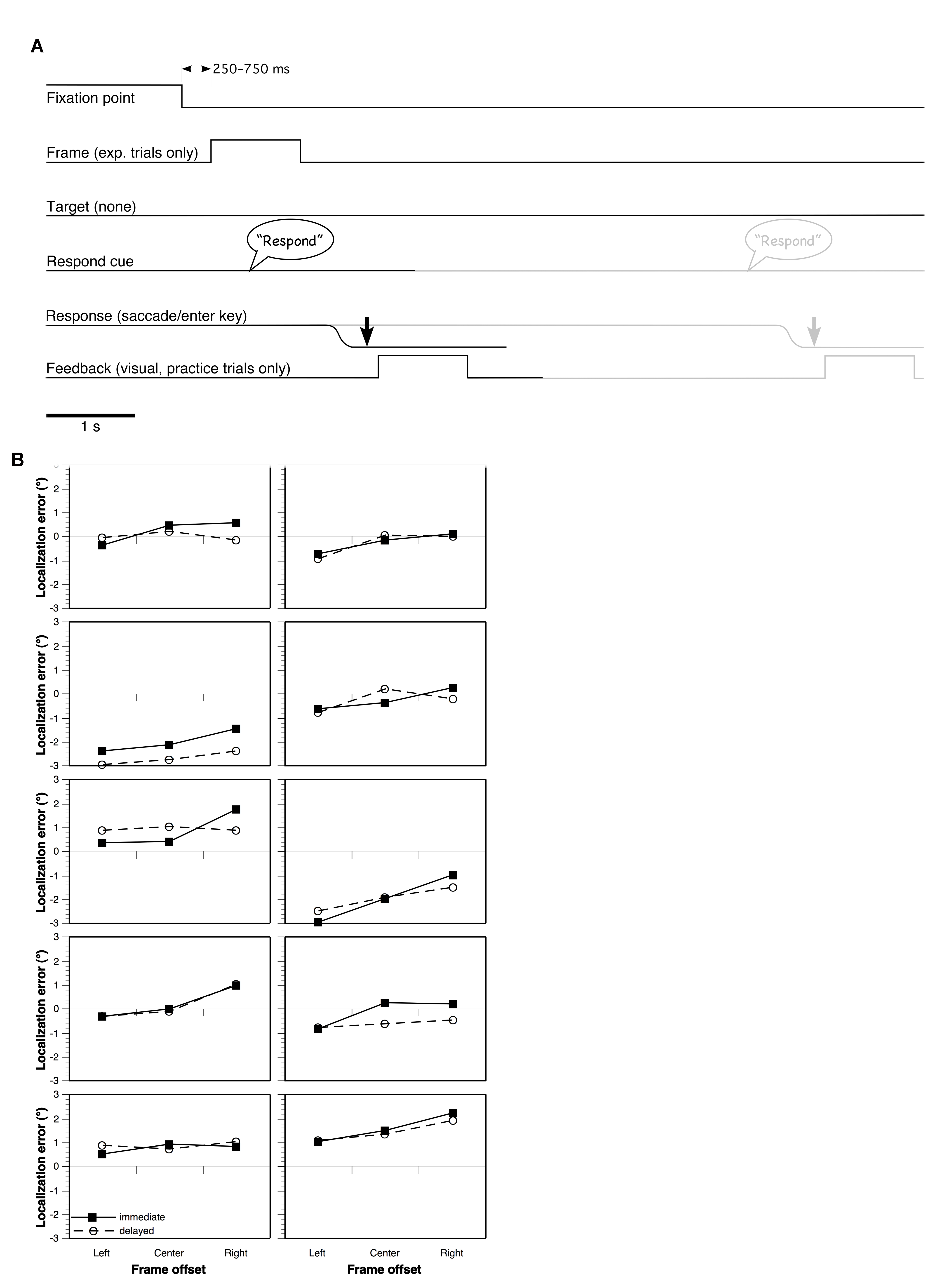

Supplement: Figure S4 — (A) Time line of task events for immediate (black) and delayed (gray) sensorimotor responses toward the apparent midline. (B) An inverse Roelofs effect for immediate (solid line) and delayed (dashed line) sensorimotor responses toward the apparent midline, for each of ten subjects. (856 KB TIF). [file pbio.0020364.sg004.tif]

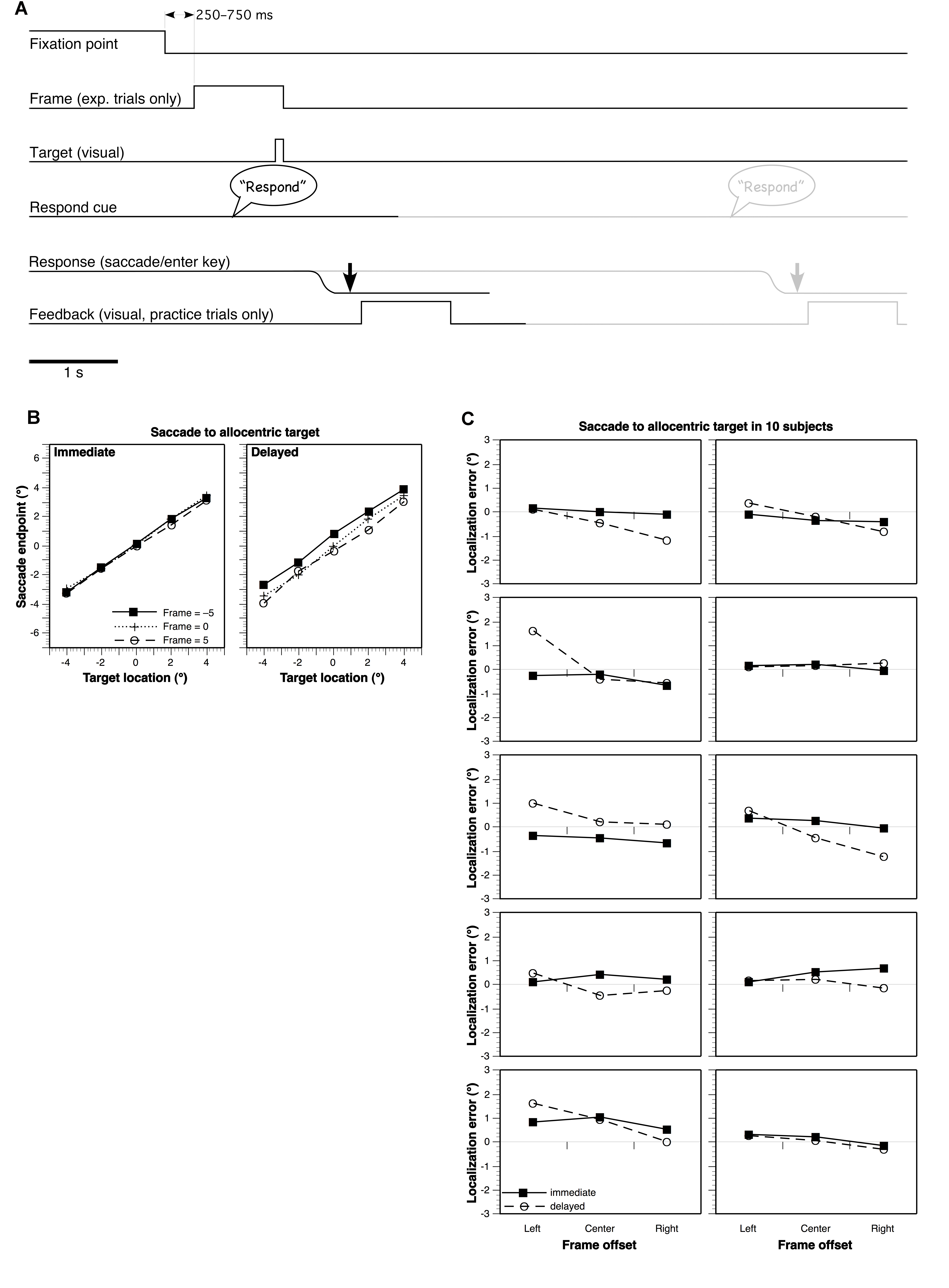

Supplement: Figure S6 — (A) Time line of task events for immediate (black) and delayed (gray) sensorimotor responses to targets defined allocentrically. (B) Effect of frame offset on immediate (solid line) and delayed (dashed line) sensorimotor responses to targets defined allocentrically, for each of five target locations. (C) Effect of frame offset on immediate (solid line) and delayed (dashed line) sensorimotor responses to targets defined allocentrically, for each of ten subjects. (1.8 MB TIF). [file pbio.0020364.sg006.tif]
